# Aetiologies of profound bilateral sensorineural hearing loss among children in Ekiti State, South Western Nigeria

**DOI:** 10.11604/pamj.2021.38.98.21438

**Published:** 2021-01-28

**Authors:** Oyebanji Anthony Olajuyin, Oladele Simeon Olatunya, Toye Gabriel Olajide, Ademola Busayo Olajuyin, Adebola Ayotomiwa Olajuyin, Adefunke Olarinre Babatola, Akinwumi Kolawole Komolafe

**Affiliations:** 1 Department of Ear, Nose and Throat, Ekiti State University Teaching Hospital, Ado Ekiti, Ekiti State, Nigeria,; 2 Department of Paediatrics, Ekiti State University Teaching Hospital, Ado Ekiti, Ekiti State, Nigeria,; 3 Department of Ear, Nose and Throat, Federal Teaching Hospital, Ido Ekiti/Afe Babalola University, Ado Ekiti (ABUAD), Ekiti State, Nigeria,; 4 Department of Family Medicine, Ekiti State University Teaching Hospital, Ado Ekiti, Ekiti State, Nigeria,; 5 Department of Obstetrics-gynaecology, Ekiti State University Teaching Hospital, Ado Ekiti, Ekiti State, Nigeria

**Keywords:** Profound bilateral, sensorineural, hearing loss, aetiologies, unknown, measles, immunization

## Abstract

**Introduction:**

a strong need exists for the knowledge of aetiologies of diseases as this will guide the clinicians on the strategies for their treatment and prevention. In this study, we determined the aetiologies of profound bilateral sensorineural hearing loss (pbSNHL) with a view to provide the relevant data needed for preventive and therapeutic intervention among children in Ekiti State, South Western Nigeria.

**Methods:**

medical records of children with pbSNHL seen over a ten-year period were analysed.

**Results:**

in all, records of 142 children with pbSNHL were analysed. The results showed spectrum of aetiologies that were similar to those reported decades ago with the 'unknown' assuming a recurring decimal. Of the known (acquired) cases, measles takes up the largest 'chunk' with a prevalence of 45.8%. Twenty-eight (32.2%) of those with febrile illnesses had treated their fever with ototoxic antibiotics. Late diagnosis was characteristic of the pbSNHL.

**Conclusion:**

this study shows that there hasn't been any significant shift in the common causes of pbSNHL. Of great concern is the persistence of the 'unknown' causes which assumes a recurring decimal in this and previous studies. Also worrisome is the high prevalence of measles-induced pbSNHL despite the availability of anti-measles vaccine. We therefore suggest effective immunization against measles and other vaccine-preventable causes of pbSNHL. The need for intensive research on the unknown causes of pbSNHL is hereby stressed. Also recommended is routine hearing assessment for survivors of febrile conditions so as to detect, if any, hearing loss promptly.

## Introduction

Profound bilateral sensorineural hearing loss (pbSNHL) in children is an otologic tragedy. Apart from being a hidden disability which makes early diagnosis difficult, the condition is largely irreversible and without rehabilitation, the victim risks being deaf and dumb. In Nigeria, the affected children often suffer untold hardship, negligence and isolation. Sometimes, the grown-up victims become destitutes and resort to street begging. Even those who are institutionalized in the school for the deaf are not fully integrated with members of the society. Citing Holborow *et al*. Ibekwe noted that up to 500,000 children in our environment are profoundly deaf at the time of their report [1]. Smith and Hatcher reported a prevalence of 2.7 per 1000 of severe to profound hearing loss among 2-10-year-old children in all villages of 8 districts of the Gambia [2]. In Eastern Finland, a prevalence of 2.1 per 1000 live births of bilateral sensorineural hearing impairment was recorded among children born in 1974-1987 [3].

In his own report, James Coplan said, 1 child per 1000 is born deaf and additional 2 are deafened during childhood [4] while Naarden found annual prevalence of moderate to profound hearing loss, 90% of which were sensorineural to be 1.1 per 1000 [5]. The aetiologies of sensorineural hearing loss vary in distribution from place to place. In Finland, the aetiologies of bilateral sensorineural hearing impairment were classified as: unknown 30%; genetic 41%; congenital nongenetic 13% and delayed-onset nongenetic 16% [3]. The distribution of deafness in Afyon school for the deaf in Turkey were: febrile convulsion 26.9%; unknown 26.1%; hereditary 23.8; meningitis 10%; measles 6.1% and miscellaneous 6.6% [6]. Ibekwe, in Enugu, Nigeria, reported the causes as: unknown 20.6%; hereditary 6.4%; febrile illnesses 41.3%; meningitis 12.7%; measles 7.9%; mumps 6.4% and whooping cough 3.2% [1]. Also in Ilorin, Nigeria, Dunmade *et al*. found: unknown 34.8%; febrile illness 18.3%; measles 13.9%; meningitis 8.7%; mumps 6.9% and severe birth asphyxia 4.3% as the main differential diagnosis of profound bilateral sensorineural hearing loss [7].

The aetiologies of sensorineural hearing loss could be identified prenatally. According to Glenn Isaacson, advances in the field of antenatal diagnosis have made possible the detection of profound sensorineural hearing loss prior to birth [8]. Also, there are postnatal means to identify congenital and acquired sensorineural hearing loss. However, at whatever stage the diagnosis is made, the fundamental tool of medical diagnosis is 'history-taking'. As noted by Ozel *et al*. the cause of hearing loss is often understood from medical history [9]. Also noted by Chen and Oghalai: “careful history-taking can reveal the etiology for congenital hearing loss, especially non-genetic congenital hearing loss” [10]. Thus, history-taking remains fundamental to the identification of pbSNHL. History-taking of pbSNHL among children may involve the parents, guardians, teachers and or eyewitness accounts. The history, if well taken is expected to reveal details of events in chronological order. This is generally complemented with thorough physical examination. Further evaluation is conducted with the use of ancillary investigations. These may include microbiological, haematological, biochemical, histopathological, immunological, radiological, genetic and audiometric tools of medical diagnosis. However, the choice and extent of investigations depends on the clinical suspicion and availability of the relevant ancillary tools.

A major challenge to accurate diagnosis of pbSNHL in our own setting however, is failure to recall all the relevant diagnostic information. This, often, is due to late presentation which has been attributed to failure of parents to seek early otolaryngologic help for their deaf children [7]. Also, a possible cause of the delayed presentation is the cryptic nature of hearing loss. As known, hearing loss is a hidden disability that makes prelingual detection impossible unless a high index of suspicion or deliberate screening exists. Without such practice, early diagnosis, rehabilitation and prevention of pbSNHL would be almost impossible. Thus, a high index of suspicion or screening based on knowledge of the aetiological factors are important for early diagnosis, management and prevention among children. It is the need to enhance knowledge of the aetiologies of early diagnosis that this study was conducted.

## Methods

Study setting: this study was conducted at the otolaryngological and paediatric clinics of two tertiary hospitals in South Western Nigeria between January 2009 to December 2018. The hospitals provide specialist care for the host community and neighbouring states. Study design: children diagnosed with profound bilateral sensorineural hearing loss in otolaryngologic and paediatric clinics were retrospectively studied for the causes of their hearing loss. The tools of investigations were records of their illnesses retrieved from the central and peripheral medical information units of our hospitals. Information retrieved were socio-demographic data of parents/guardians, immunization status of the children, age of speech acquisition, onset of hearing loss, onset of speech loss, past medical history, pregnancy and birth history, records of otoscopic examinations, audiological assessment (tympanometry, pure tone audiometry (PTA), otoacoustic emission (OAE), auditory brainstem response (ABR) and treatments previously received.

The degree of hearing loss was classified based on the World Health Organsation as mild (26 - 40db), moderate (41 - 60db), severe (61 - 80db) and profound (>80db). Exclusion criteria: excluded were cases of isolated conductive hearing loss, unilateral hearing loss, mild or moderate hearing loss. Ethical considerations: the study was approved by the institution's ethics and research committee. Data analysis: the data generated were entered into personal computer and simple descriptive statistics was performed using SPSS version 14.

## Results

**Figure 1:**
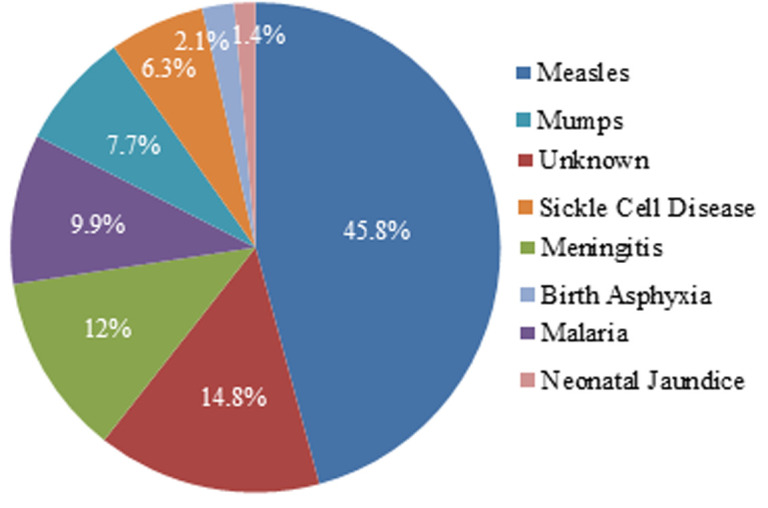
percentage distribution of the various aetiological factors of profound sensorineural hearing loss in the study locale

In all, records of 142 children were analysed. They were aged 6 to 15 years. There were 73 males and 69 females (Table 1), giving a M: F ratio of 1: 1.1. The following (Figure 1) were the aetiological factors of pbSNHL in the study locale: unknown, measles, meningitis, malaria, mumps, sickle cell disease (SCD), birth asphyxia and neonatal jaundice. Twenty-eight (32.2%) of those with febrile illness-induced pbSNHL used ototoxic antibiotics for the febrile illnesses. Immunization was received by 13 (20%) of those with measles-induced hearing loss. The duration between onset of hearing loss and consultation with otolaryngologists ranged from 2 years to 7 years. Some (29.6%) of the patients were pupils from the school for the deaf.

**Table 1:** age versus gender distribution of the patients

Age (years)	Male (%)	Female (%)	Total (%)
6 - 7	18 (12.7%)	20 (14.1%)	38 (26.8%)
8 - 9	17 (12.0%)	25 (17.6%)	42 (29.6%)
10 - 11	10 (7.0%)	13 (9.2%)	23 (16.2%)
12 - 13	16 (11.3%)	7 (4.9%)	23 (16.2%)
14 - 15	12 (8.4%)	4 (2.8%)	16 (11.2%)
Total	73 (51.4%)	69 (48.6%)	142 (100%)

## Discussion

Profound bilateral sensorineural hearing loss (pbSNHL) is an otologic tragedy. The condition constitutes huge educational, social and economic liability for individual, families and the society at large. The present study focused on the aetiologies and distribution of pbSNHL among children in Ekiti State, South Western Nigeria. The results showed a spectrum of aetiologies similar to those reported by other scholars decades ago in the West Africa subregion [7,11-13]. Thus, no significant shift in aetiology had occurred between now and then in the study setting. Of great concern however, was the occurrence of unknown causes of pbSNHL in this and previous studies. Although, the 14.8% reported in this study is a far cry from what was reported in other studies [7,11,12], the recurring nature of the unknown cases constitutes a great clinico-epidemiological challenge. One reason that might be advanced for the recurring 'decimal' is: inability of pregnant women to recognise the characteristic features of some intrauterine causes of pbSNH. A typical example is the occurrence of maternal rubella, the features of which may appear a red herring. As noted by Ibekwe, women who bring their children to the clinic might not understand what you are referring to when you try to describe to them the nature of rubella [1]. Thus, when such cases are brought to the clinic, they are likely to be classified as 'unknown' or 'idiopathic'. Also, to be factored-in, is the inadequate diagnostic facilities particularly in our own setting that may limit proper diagnosis as observed by Chukuezi [14]. Furthermore, the lack of pre-natal diagnosis may reduce the diagnostic yield thereby raising the bar for the unknown cases of pbSNHL. To improve the diagnostic yield therefore, there is the need to sensitize mothers towards having a high index of suspicion of intrauterine causes of pbSNH and the role of history-taking in clinical diagnosis must not be compromised. Also, the provision of specific diagnostic facilities and more researches on the prenatal diagnosis of congenital sensorineural hearing loss may scale down the records of unknown causes of pbSNHL among children.

Ranking highest among the known causes of pbSNHL in this study was measles with a prevalence of 45.8%. This was recorded in some other studies [12]. However, in other studies [1,7], the show of strength by measles was dwarfed by other aetiological factors. Although the reasons for this discordance could not be established, it could be a reflection of the differences in study settings and immunization coverage of children in the different localities. As revealed by Wright and Leigh, the incidence of measles-induced sensorineural hearing loss is inversely proportional to increased immunization coverage of children [12]. Thus, the prevalence of measles-induced pbSNHL in a community could be an index of immunization coverage of the community. Paradoxically however, 20% of the children with measles-induced pbSNHL in this study had previously been immunized against measles. Although, it could not be ascertained if this was due to primary or secondary vaccine failure, the occurrence of measles vaccine failure had previously been reported [15,16]. This is attributed to the use of non-potent vaccines (failed cold chain), inappropriate dosing and malnutrition [15-17]. Thus, there is the need for a paradigm shift from quantitative to qualitative immunization of children against measles in compliance with the best international practice. Researches into a cost-effective means of ascertaining 'on-the-spot' seroconversion among the immunised children is a worthwhile efforts. In this regard, the authors propose a future research into the on-the-spot use of non-invasive techniques such as digital wrist sensors to detect anti-measles specific immunoglobulin similar to that proposed for myocardial diseases [18] after immunization.

Next in ranking among the acquired causes of pbSNHL in this study is meningitis. Our records showed that meningitis was responsible for 17 (12.0%) of the cases in this study. This is proportionally similar to, though numerically different from the 33 (11%) of the 298 profoundly deaf children recorded by Ijaduola in Lagos, Nigeria. Further analysis of our own series showed that: in 23.5% of the meningitic cases, chronic suppurative otitis media (CSOM) was the focus of infection. Although, there was no active ear disease at the time of review, this finding underscores the need to pay close attention to CSOM in children as studies have shown that it could be due to parental negligence. As noted by Okafor, chronic suppurative otitis media is a complication, often due to negligence, of the acute variety. As he put it, acute otitis media are often delayed while looking around for a local remedy [19]. Also, there was a report that ear discharge is believed by mothers to be a normal feature of teething hence innocuous [20]. This myth might prevent early uptake of treatment for ear discharge among children. Thus, there is the need to sensitize mothers on prompt treatment of ear discharge among children. This may reduce the incidence of pbSNHL consequent upon reduction in the incidence of CSOM-induced meningitis.

Surprisingly, malaria, a very common cause of febrile illness in Nigeria was responsible for only 14 (9.9%) of the cases in this study. This finding is at variance with the 26 (41.3%) reported by Ibekwe [1]. The role of malaria in the aetiopathogenesis of pbSNHL has not been fully established. In the view of Dunmade *et al*. malaria infection may cause deafness either by local action with microvascular changes in the end arteries of the cochlea or in a general way by lowering resistance to disease and thus enhancing the adverse effects of other infections. Although, high fever which is characteristics of most malaria infection in children, may damage the sensitive hair cells of the inner ear, the clogging of inner ear micro vessels similar to what is suggested in sickle-cell induced SNHL seems to be more relevant. This is supported by finding pbSNH in 6.3% of patients with sickle cell disease in this and bilateral profound sudden sensorineural hearing loss in a child with sickle cell anaemia in Mace *et al*. [21] studies. The impacts of recurrent vaso-occlusion that may result in labyrinthine haemorrhage (LH) and/or labyrinthitis ossificans (LO) has been suggested as the mechanism of pbSNHL in SCD [22-24]. Furthermore, the roles of anemia as the possible pathophysiologic mechanism have also been highlighted [22]. Overall, there is a general consensus that cochlear blood flow changes are fundamental to inner ear pathology such as sensorineural hearing loss seen in SCD. These hypotheses are further supported by the observation of the fact that the inner ear structures are mainly supplied by end arteries [22], hence bereft of collateral circulation. When such end arteries are blocked, the outcome is ischemia and damage of the target organs.

Mumps was recorded in 11 (7.7%) of the cases in this series. The most common manifestation was bilateral parotitis. This might blur a co-existing sensorineural hearing loss. A high index of suspicion of otologic involvement is therefore important to clinch a diagnosis. Birth asphyxia (BA) and severe neonatal jaundice (SNNJ) were non-infectious disorders recorded as causes of pbSNHL in this study. They contributed 2.1% and 1.4% prevalence rate respectively. They were diagnosed based on data from pregnancy and birth records. Although, advances in the field of antenatal diagnosis have made possible the detection of profound sensorineural hearing loss prior to birth [8], such was not feasible in our own study because of its retrospective nature. For this reason, we could not differentiate pbSNHL of peri or neonatal from antenatal causes of pbSNH. However, it is our hope that future studies on these culprits will address this issue. This limitation notwithstanding, the very low prevalent rate of BA and SNNJ-induced pbSNHL in this study points to improved maternal and child-care which if sustained can reduce the prevalence of pbSNHL through a well co-ordinated maternal and child health care.

Another major finding in this study is the abuse of ototoxic antibiotics by patients with infection-induced pbSNHL. Some patients with measles, meningitis and other febrile illnesses were noted to have used and abused ototoxic antibiotics without doctors` prescription. In such circumstances, it becomes a diagnostic conundrum to differentiate between ototoxicity and the febrile causes of the auditory shutdown. Ignorance, poverty and the ease with which antibiotics can be bought over the counter without appropriate prescription have been identified as the main cause of antibiotic abuse in our own setting. Health education and improved socio-economic wellbeing are therefore important to prevent pbSNHL caused by the abuse of ototoxic antibiotics. Periodic serum assay of ototoxic antibiotics used in those on hospital admission is equally important to keep the drugs within the safety margin. Also, there is the need for the government agency to enforce the rules guiding the sales of on the counter antibiotics among the general public.

Important to note in this study is the late diagnosis of pbSNHL. As observed, the patients were seen between 2-7 years after the onset of the pbSNHL. Although, this could be attributed to poor access to otolaryngologists, the failure to seek early otolaryngologic help is a major contributing factor. According to Ijaduola, one reason for late diagnosis is because mothers delay seeking help; they wait until their children have failed to acquire speech and even when speech has failed to develop, they delay further with various excuses ranging from having late starters in their family or the child having “only tongue tie” [11]. Given these parental negligence and lackadaisical attitude towards otologic care, there is the need to sensitize mothers on the implications of pbSNHL and failure to heed the advice on the prevention or management of the condition among children. Also, routine hearing assessment following recovery from febrile illnesses such as measles, meningitis and malaria may fastrack the diagnosis and rehabilitation of children with pbSNHL.

## Conclusion

This study shows that there hasn't been any significant shift in the common causes of pbSNHL. Of great concern is the persistence of the 'unknown' causes which assumes a recurring decimal in this and previous studies. Also worrisome is the high prevalence of measles-induced pbSNHL despite the availability of anti-measles vaccine. We therefore suggest effective immunization against measles and other vaccine-preventable causes of pbSNHL. The need for intensive research on the unknown causes of pbSNHL is hereby stressed. Also recommended is routine hearing assessment for survivors of febrile conditions so as to detect, if any, hearing loss promptly.

### What is known about this topic

- Aetiologies of profound bilateral sensorineural hearing loss could be congenital or acquired;
- Some of the acquired varieties can be prevented by immunization.

### What this study adds

- Chronic suppurative otitis media is a risk factor in profound bilateral sensorineural hearing loss;
- Need for routine hearing assessment of survivors of febrile condition.
